# Validation of Point-of-Care Ultrasound to Measure Perioperative Edema in Infants With Congenital Heart Disease

**DOI:** 10.3389/fped.2021.727571

**Published:** 2021-08-23

**Authors:** Jessica N. Persson, Jacqueline Holstein, Lori Silveira, Aimee Irons, Taufiek Konrad Rajab, James Jaggers, Mark D. Twite, Carly Scahill, Mary Kohn, Christine Gold, Jesse A. Davidson

**Affiliations:** ^1^Department of Pediatrics, University of Colorado Anschutz, Aurora, CO, United States; ^2^Heart Institute, Children's Hospital Colorado, Aurora, CO, United States; ^3^Research Institute, Children's Hospital Colorado, Aurora, CO, United States; ^4^Section of Congenital Heart Surgery, University of Colorado Anschutz, Aurora, CO, United States; ^5^Department of Anesthesiology, University of Colorado Anschutz and Children's Hospital Colorado, Aurora, CO, United States

**Keywords:** point-of-care ultrasound, congenital heart disease, fluid overload, cardiac surgery, edema

## Abstract

**Purpose:** Fluid overload is a common post-operative issue in children following cardiac surgery and is associated with increased morbidity and mortality. There is currently no gold standard for evaluating fluid status. We sought to validate the use of point-of-care ultrasound to measure skin edema in infants and assess the intra- and inter-user variability.

**Methods:** Prospective cohort study of neonates (≤30 d/o) and infants (31 d/o to 12 m/o) undergoing cardiac surgery and neonatal controls. Skin ultrasound was performed on four body sites at baseline and daily post-operatively through post-operative day (POD) 3. Subcutaneous tissue depth was manually measured. Intra- and inter-user variability was assessed using intraclass correlation coefficient (ICC).

**Results:** Fifty control and 22 surgical subjects underwent skin ultrasound. There was no difference between baseline surgical and control neonates. Subcutaneous tissue increased in neonates starting POD 1 with minimal improvement by POD 3. In infants, this pattern was less pronounced with near resolution by POD 3. Intra-user variability was excellent (ICC 0.95). Inter-user variability was very good (ICC 0.82).

**Conclusion:** Point-of-care skin ultrasound is a reproducible and reliable method to measure subcutaneous tissue in infants with and without congenital heart disease. Acute increases in subcutaneous tissue suggests development of skin edema, consistent with extravascular fluid overload. There is evidence of skin edema starting POD 1 in all subjects with no substantial improvement by POD 3 in neonates. Point-of-care ultrasound could be an objective way to measure extravascular fluid overload in infants. Further research is needed to determine how extravascular fluid overload correlates to clinical outcomes.

## Introduction

Fluid overload, defined as excess intra- and/or extravascular fluid in the body, is associated with increased morbidity and mortality in critically ill children (1–3). The etiology of fluid overload is multifactorial and includes fluid retention due to neurohormonal pathway activation, congestive heart failure, iatrogenic fluid administration, and capillary leak (4–6). One group at high risk of developing fluid overload is pediatric patients with congenital heart disease (CHD) undergoing cardiac surgery (7). Fluid overload is prevalent in this population, with rates ranging from 30–100% depending on the degree of fluid overload evaluated and method of assessment (1, 8, 9). Cardiopulmonary bypass itself directly causes fluid overload as well as other physiologic derangements that further exacerbate fluid overload, such as ischemia-reperfusion injury, systemic inflammation, capillary leak, and low cardiac output syndrome (10–12).

Fluid overload has detrimental effects on the body. Intravascular fluid overload elevates central venous pressure (CVP) leading to poor renal perfusion and subsequent acute kidney injury (AKI). Extravascular fluid overload, also known as edema, compromises abdominal and thoracic compliance thereby impeding ventilation, and negatively impacts oxygenation, cardiac compliance, and intestinal function. As a result, post-operative fluid overload following cardiac surgery in pediatric patients, especially infants, is associated with significant morbidity including AKI, longer mechanical ventilation dependence, prolonged length of stay, and increased mortality (3, 7, 8, 12–14). Therefore, early detection and management of fluid overload is crucial in neonates and infants following cardiac surgery given the negative impact of fluid overload on patient outcomes. Unfortunately, fluid overload is difficult to accurately quantify in pediatric patients. The most utilized methods for assessing fluid overload include trending daily weights, net fluid balance, physical examination findings, and CVP measurements, all of which can be imprecise and can falsely represent a patient's fluid status due to the inherent inaccuracies in data collection and challenges distinguishing between intra- and extra-vascular fluid overload (4, 15–17). Ultrasound, a fast and painless imaging modality that uses benign sound waves instead of ionizing radiation, has been studied in adults as a non-invasive measurement of fluid status by assessing the distensibility of vascular structures or extravascular fluid accumulation (18–26). MuscleSound, Inc., a Denver based ultrasound software company, has recently developed automated ultrasound measurements of skeletal muscle layers in the adult population that could be used to measure post-operative changes in subcutaneous edema and extravascular fluid overload (27–30).

While the use of ultrasound to measure body edema is promising, no studies have assessed this ability in pediatric or adult populations with chronic medical conditions such as CHD who commonly struggle with fluid overload. Therefore, we aimed to use ultrasound for the novel purpose of quantifying edema in a high-risk clinical population, specifically neonates and infants undergoing CHD surgery. Additionally, we partnered with MuscleSound, Inc. to pilot their automated ultrasound measurement technology.

In this study, we sought to validate the use of point-of-care ultrasound to measure skin edema in neonates and infants with CHD. Our secondary aim was to perform pilot testing of MuscleSound, Inc.'s automated measurement software for skin ultrasound images. We utilized a clinically available point-of-care ultrasound system paired with proprietary MuscleSound, Inc. software to measure the subcutaneous tissue thickness in infants with CHD, in order to quantify changes induced by cardiac surgery and differences compared to healthy, age-matched controls. We hypothesized that ultrasound could detect and quantify changes in subcutaneous tissue depth induced by postoperative edema with high intra- and inter-user reliability. The possibility to quantify edema with a reliable, automated, non-invasive, bedside tool could provide much needed insight into a patient's fluid status, especially in neonates and infants with CHD who have many risk factors for extravascular fluid overload but whose fluid status can be difficult to accurately assess.

## Materials and Methods

We performed a prospective observational cohort study of neonates and infants undergoing cardiothoracic surgery and healthy controls. The primary aim of the study is to validate the use of point-of-care ultrasound for the measurement of subcutaneous tissue thickness in infants with CHD and neonates with and without CHD. The secondary aims were to (i) assess inter- and intra-user variability of the point-of-care skin ultrasound, (ii) compare subcutaneous tissue thickness in pre-operative neonates with CHD to healthy, age-matched controls, (iii) compare acute postoperative changes in subcutaneous tissue thickness as a measurement of edema in neonates and infants with CHD, and (iv) pilot test the use of MuscleSound, Inc.'s automated point-of-care ultrasound software for the measurement of subcutaneous tissue thickness in neonates and infants.

Control subjects were enrolled at University of Colorado Hospital's Well Baby Nursery between December 2019 and November 2020. Surgical subjects were enrolled at the Heart Institute at Children's Hospital Colorado between January 2020 and February 2021. All surgical subjects underwent cardiac surgery at Children's Hospital Colorado, a quaternary care center, and received post-operative care in the pediatric cardiac intensive care unit. The study protocol was approved by the Colorado Multiple Institutional Review Board. Parental written informed consent was obtained for all subjects.

Control subjects were enrolled if they were healthy term neonates (less than or equal to 30 days old at the time of enrollment). Control subjects with a previous history of surgery with general anesthesia, renal dysfunction, or prematurity (<36 weeks corrected gestational age) were excluded as these factors could confound the subject's fluid status. Surgical subjects were enrolled if they were <12 months old at the time of enrollment. Surgical subjects were included if they had hemodynamically significant CHD and were undergoing cardiac surgery, with or without cardiopulmonary bypass, to repair or palliate their congenital heart defect. Exclusion criteria for surgical subjects included: corrected gestational age <36 weeks at time of enrollment and/or known renal dysfunction.

Baseline characteristics were collected for all subjects, including demographics and weight, at enrollment. For surgical subjects, operative data was acquired, which included cardiac diagnoses, type of cardiac surgery, cardiopulmonary bypass time, and Society of Thoracic Surgeons-European Association for Cardio-Thoracic Surgery (STAT) score (31).

Point-of-care ultrasound images were obtained using the following tools: Lumify by Philips broadband linear array transducer (model L12-4), Philips Lumify Ultrasound application (software version: 3.0 453562091941 20-10-09), and Samsung Galaxy Tab A tablet (model number SM-T510). For each subject, ultrasound of the skin layers was obtained on the right and left sides of four body sites. These four ultrasound sites included: upper anterior chest (at the mid-clavicular line, one centimeter above the nipple), lateral chest (at the mid-axillary line, at the level of the nipple), lateral abdomen (at the mid-axillary line, at the level of the umbilicus), and anterior thigh (midline, approximately two centimeters above the patella). Three ultrasound images were obtained at each site in order to assess intra-user variability. Two trained research members performed the ultrasound at each body site for each subject so that inter-user variability could be assessed. For control subjects, the ultrasound was performed at one time point, specifically at time of enrollment, to allow for baseline comparison to pre-operative surgical subjects. For surgical subjects, the baseline ultrasound was performed pre-operatively at time of enrollment and post-operative ultrasounds were performed daily from post-operative day (POD) 1 to POD 3. If the surgical subject had an open chest post-operatively, the daily ultrasounds were extended through POD 5.

Following completion of the daily ultrasound, the ultrasound images were exported to MuscleSound, Inc.'s secure cloud-based server. MuscleSound, Inc. provided scaled ultrasound images from which the subcutaneous tissue thickness for each ultrasound image was manually measured. Two trained research members manually measured the same images for ~10% of the total images to ensure there was minimal variability in measurements. The remaining images were manually measured by one of the two trained research members. Automated measurements for the right-sided lateral abdomen and left-sided anterior thigh images were performed by the MuscleSound, Inc. team using their proprietary software. MuscleSound, Inc. analyzes greyscale ultrasound images for various patented applications. MuscleSound, Inc.'s automated image processing algorithms were extended to identify tissue structures via high contrast filters and then select the tissue of interest, specifically the adipose-muscle boundary for this study. The subcutaneous tissue depth was then determined as distance from probe on skin to the adipose-muscle boundary in millimeters.

After ~50% of control and surgical subject enrollment, an interim qualitative assessment of the images was performed. The goals of this interim analysis were to evaluate image quality and allow time for adjustment of MuscleSound, Inc.'s automated measurement software. After this interim analysis, all research team members completed a short ultrasound training on how to optimize ultrasound images at each site with the goal to improve image quality. This interim ultrasound training specifically focused on identifying ultrasound landmarks for each body site and centering the area of interest within the frame of the ultrasound image. Additionally, we created a short handout of ideal image examples with labeled landmarks to have available at the bedside (Supplementary Material).

### Statistical Analysis

Baseline and surgical characteristics of the subjects were summarized using either counts (percents) for categorical data or medians (interquartile range) for continuous variables. Kruskal-Wallace tests were used to compare the three groups followed by the Wilcoxon rank sum tests for pairwise tests when the three group tests were significant (*p* < 0.05) for continuous variables and a generalization of Fisher's exact test were used to compare the three groups and pairwise comparisons for the categorical variables.

The average manual measurement from the three ultrasound images at each site was used for comparison. The right and left sides were compared using paired *t*-tests. A Student's *t*-test was used to compare the manual measurements from the baseline control subjects to the baseline surgical neonate subjects. Neonates were defined as subjects ≤ 30 days old at time of enrollment. Repeated measures ANOVA was used to compare the baseline measurements for all surgical subjects to the post-operative day measurements. The median manual measurement was compared to the automated measurement for a specific body site using the Wilcoxon signed rank test.

Intra-user variability (variability between the three images obtained at each body site by one scanner) was assessed using intraclass correlation coefficient (ICC). ICC was also used to analyze the inter-user variability (variability between the two scanners at each body site) as well as inter-user variability before and after the interim ultrasound training. For ICC, 0.75–0.9 was considered a good correlation and ≥ 0.9 was considered an excellent correlation. Correlation of the automated vs. manual measurements for the baseline right-sided lateral abdomen and left-sided anterior thigh was also performed using Pearson's product moment correlation.

## Results

### Baseline and Operative Characteristics

Fifty control subjects and 22 surgical case subjects were enrolled. Of the control subjects, all were neonates (≤ 30 days old). Of the surgical subjects, 12 were neonates and the remaining 10 were infants (between 31 days and 12 months of age). One surgical neonate withdrew on POD 1 per parent request. One surgical neonate did not have ultrasound images performed on POD 1 and 4 due to hemodynamic instability. The data and ultrasound images that were available for these two neonates were included in the analysis.

Baseline characteristics are detailed in Table 1. The cardiac diagnoses of the surgical subjects are listed in Supplementary Table 1. Operative characteristics for the surgical subjects are presented in Table 2. Neonatal surgical subjects had higher STAT category scores than the infant surgical subjects. Only neonatal surgical subjects had open chests post-operatively. Surprisingly, there were no differences in bypass time, cross-clamp time, or circulatory arrest/selective cerebral perfusion time between the neonatal and infant surgical subjects.

**Table 1:** Baseline characteristics.

**Baseline characteristics**	**Control subjects** **(*N* = 50)**	**Neonatal surgical subjects** **(*N* = 12)**	**Infant surgical subjects** **(*N* = 10)**	**Overall** ***p*** **-value** **(3-way comparison)**	**Pair-wise comparison**
**Sex**					
Male	46% (*n* = 23)^a^	50% (*n* = 6)	70% (*n* = 7)	0.42	
Gestational age (wk)	39.0 (36–42)	37.5 (36–39)	39 (35–40)	0.01	^ψ^0.0061, ^±^0.01,^€^0.46
Age at enrollment (d)	1 (1)	5.5 (4.0–6.5)	138.5 (121.0–152.0)	<0.0001	^ψ^ <0.0001, ^±^ <0.0001,^€^ <0.0001
Weight at enrollment (kg)	3.14 (2.89–3.47)	3.05 (2.75–3.35)	5.57 (4.77–6.5)	<0.0001	^ψ^ <0.27, ^±^ <0.0001,^€^ <0.0001
**Race**					
White	32 (64%)	6 (50%)	5 (50%)	0.57	
Black	7 (14%)	0 (0%)	0 (0%)	0.31	
Other	10 (20%)	2 (17%)	20 (20%)	1.0	
Unknown	1 (2%)	4 (33%)	3 (30%)	0.0009	^ψ^0.0039, ^±^0.01, 1.0
**Ethnicity**					
Not hispanic or Latino	36 (72%)	3 (25%)	5 (50%)	0.0038	^ψ^0.0031, ^±^0.04,^€^0.43
Hispanic or Latino	12 (24%)	6 (50%)	2 (20%)		
Unknown	2 (4%)	3 (25%)	3 (30%)		

*wk, weeks; d, days; kg, kilograms*.

ψ *Control compared to neonatal surgical subjects*;

± *Control compared to infant surgical subjects*;

€ *Neonatal surgical compared to infant surgical subjects*.

a *Continuous data are expressed as the median (interquartile range) and categorical data are expressed as % (n)*.

**Table 2:** Operative characteristics of surgical subjects.

**Operative characteristics**	**Neonatal surgical subjects** **(***N*** = 12)**	**Infant surgical subjects** **(***N*** = 10)**	***P*** **-value**
Single ventricle physiology	4 (33%)^a^	3 (30.0%)	1.0
STAT category			0.01
1	0 (0%)	1 (10%)	
2	1 (8%)	6 (60%)	
3	2 (17%)	2 (20%)	
4	6 (50%)	1 (10%)	
5	3 (25%)	0 (0%)	
Cardiopulmonary bypass time (min)	149 (122–165)	154 (96–183)	0.82
Cross-clamp time (min)	77 (68–87)	67 (48–105)	0.62
Deep hypothermic circulatory arrest (min)	0 (0–3)	0 (0–2)	0.97
Selective cerebral perfusion (min)	0 (0–20)	0 (0–0)	0.49
Post-operative open chest	8 (67%)	0 (0%)	0.0017

*min, minutes*.

a *Continuous data are expressed as the median (interquartile range) and categorical data are expressed as % (n)*.

### Laterality Analysis

**Figure 1:**
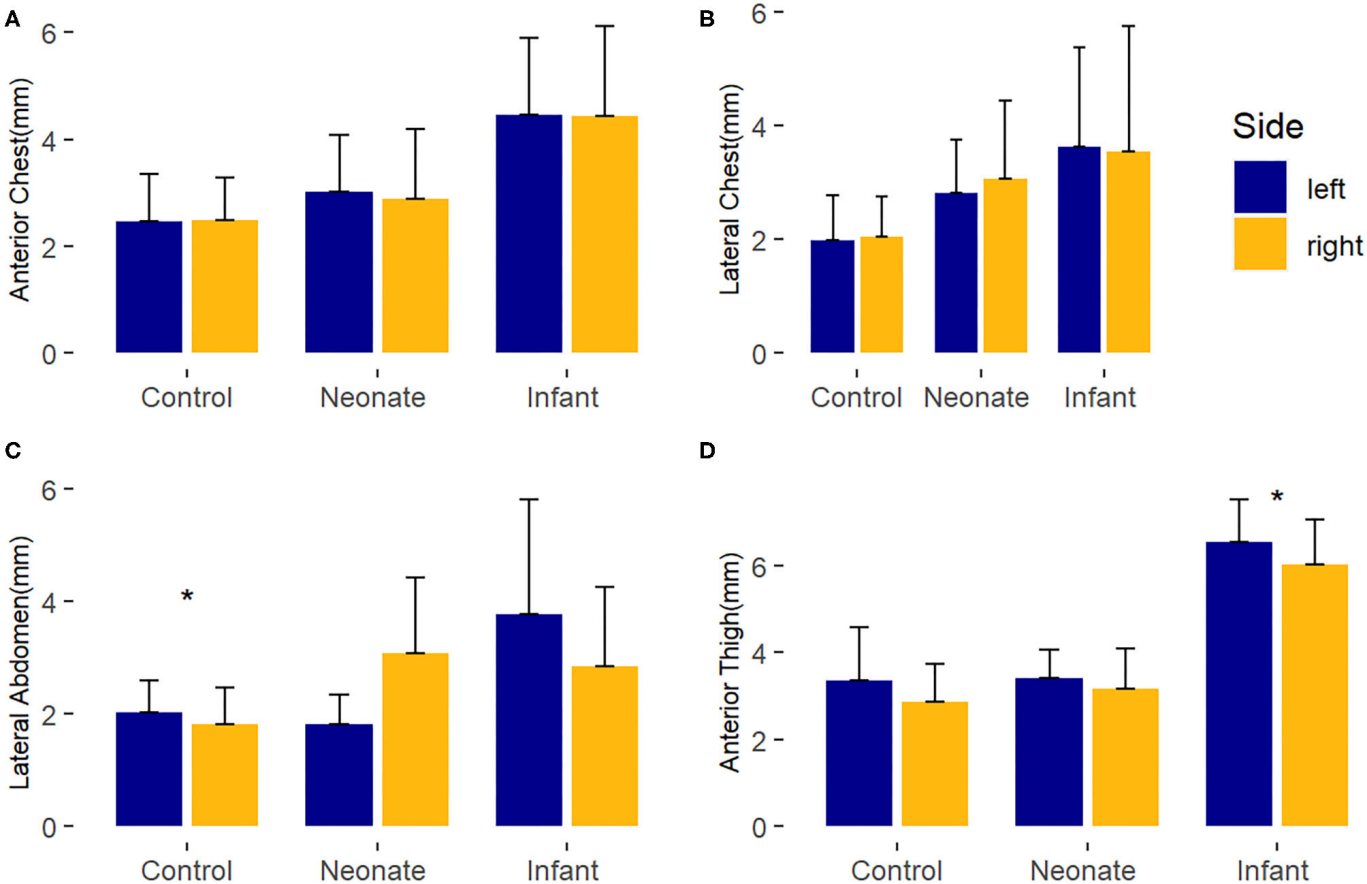
Laterality comparison of right and left-sided average baseline subcutaneous tissue thickness, by body site. ^*^, *p* < 0.05. **(A)** Anterior chest; **(B)** Lateral chest; **(C)** Lateral abdomen; **(D)** Anterior thigh.

Differences in the laterality of the body sites by ultrasound was assessed by comparing the average baseline manual measurements of the right and left sides for each body site (Figure 1). Overall, there were no major differences between the right and left sides for any group. There were two statistically significant findings of uncertain clinical significance. For control subjects, the average left lateral abdomen tissue thickness was 0.2 mm larger than right lateral abdomen (*p*-value = 0.01). In the surgical infant group, the left anterior thigh tissue thickness was 0.51 mm larger than the right anterior thigh (*p*-value = 0.04).

### Baseline Neonatal Comparison

**Figure 2:**
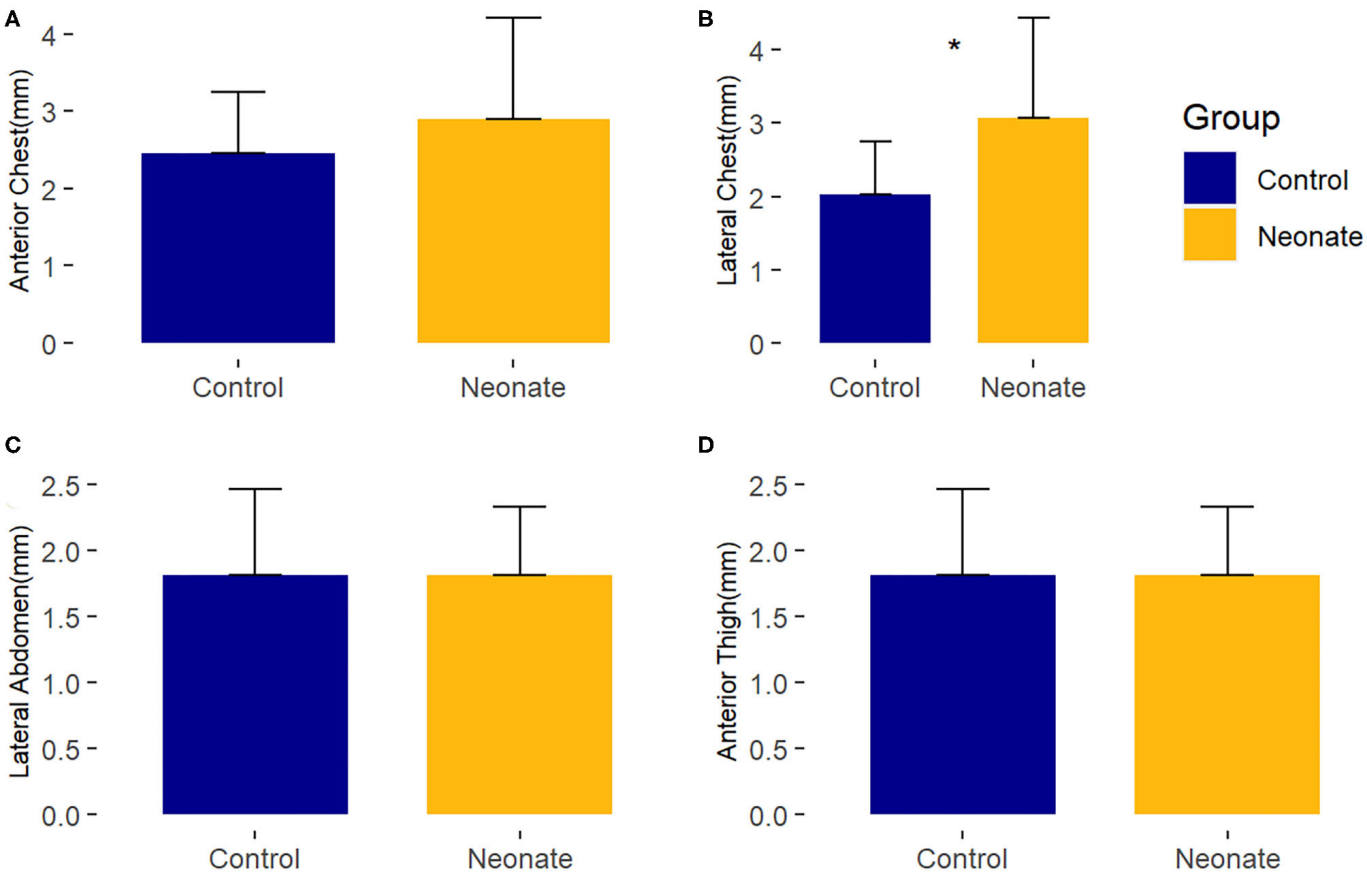
Comparison of mean baseline subcutaneous tissue thickness between controls and surgical neonatal subjects, by right-sided body site. ^*^, *p* < 0.05. **(A)** Anterior chest; **(B)** Lateral chest; **(C)** Lateral abdomen; **(D)** Anterior thigh.

Compared to healthy controls, neonates with CHD at baseline had no consistent difference in subcutaneous tissue thickness. There was a minimal (~1 mm) difference (*p*-value = 0.02) seen in the right lateral chest between the neonatal surgical subjects and control subjects of uncertain clinical significance. These findings are depicted in Figure 2 and Supplementary Table 2.

### Ultrasound Images

**Figure 3:**
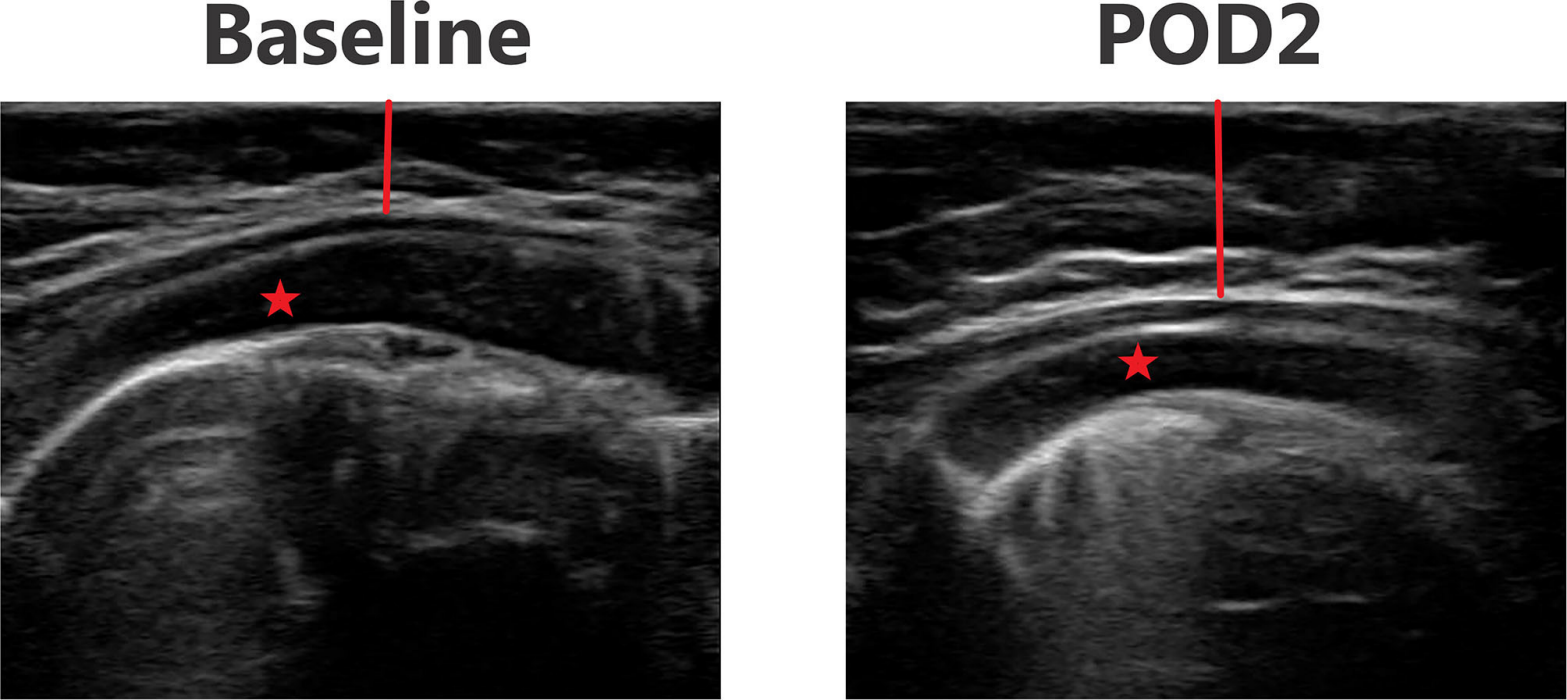
Point-of-care ultrasound images of anterior chest at baseline and POD 2 of the same patient. Both images are presented on identical scales (total depth of 2.5 cm from the skin). Red vertical line demonstrates subcutaneous tissue thickness—there is a marked increase in subcutaneous tissue seen on POD 2. Red star—pectoralis major muscle.

**Figure 4:**
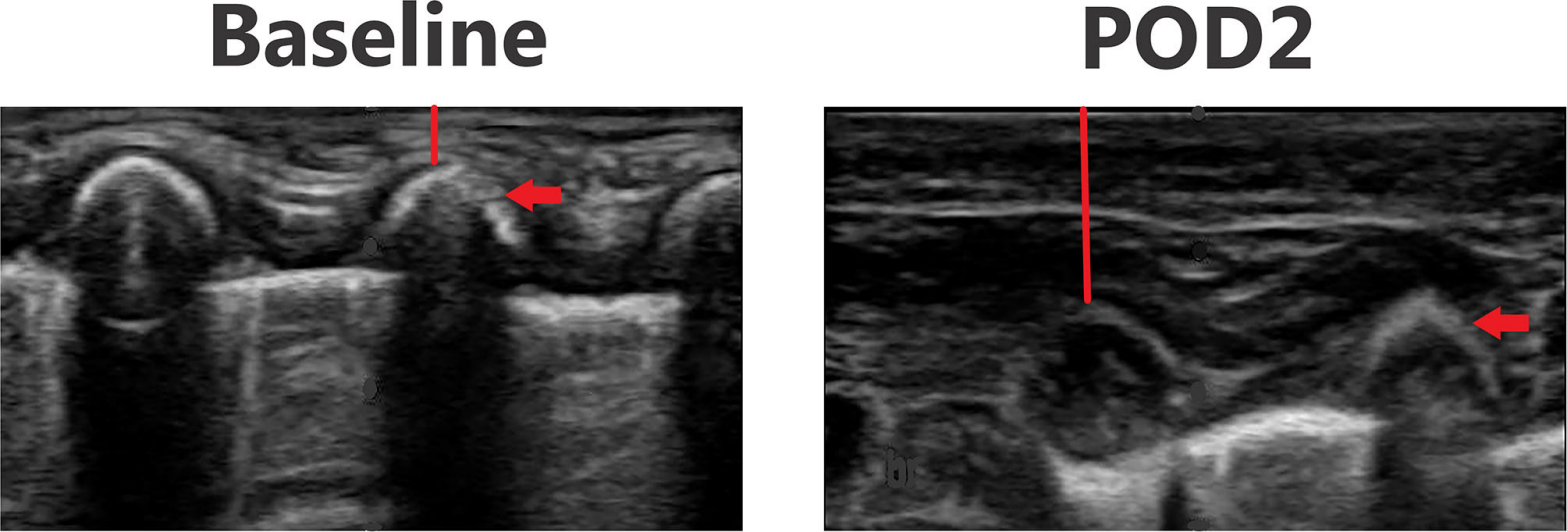
Point-of-care ultrasound images of lateral chest at baseline and POD 2 of the same patient. Both images are presented on identical scales (total depth of 1.5 cm from the skin). Red vertical line demonstrates subcutaneous tissue thickness—there is a marked increase in subcutaneous tissue seen on POD 2. Red arrow—rib bone.

**Figure 5:**
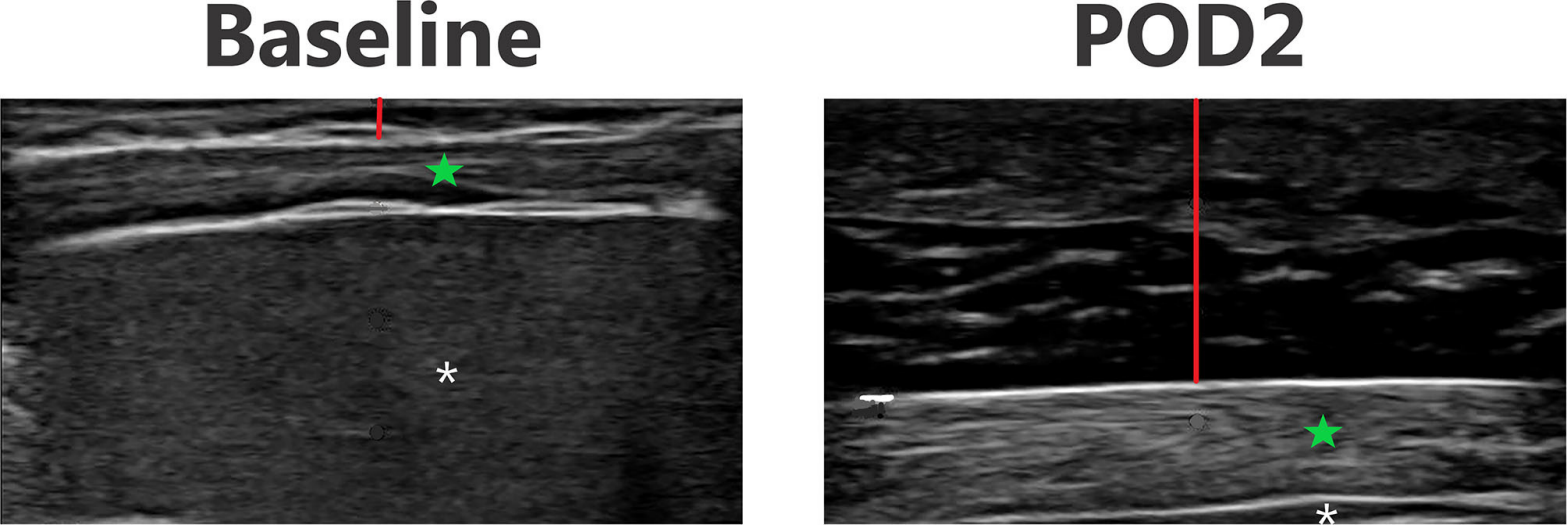
Point-of-care ultrasound images of lateral abdomen at baseline and POD 2 of the same patient. Both images are presented on identical scales (total depth of 2 cm from the skin). Red vertical line demonstrates subcutaneous tissue thickness—there is a marked increase in subcutaneous tissue seen on POD 2. Green star—echogenic muscle changes seen on POD 2. White asterisk—liver.

**Figure 6:**
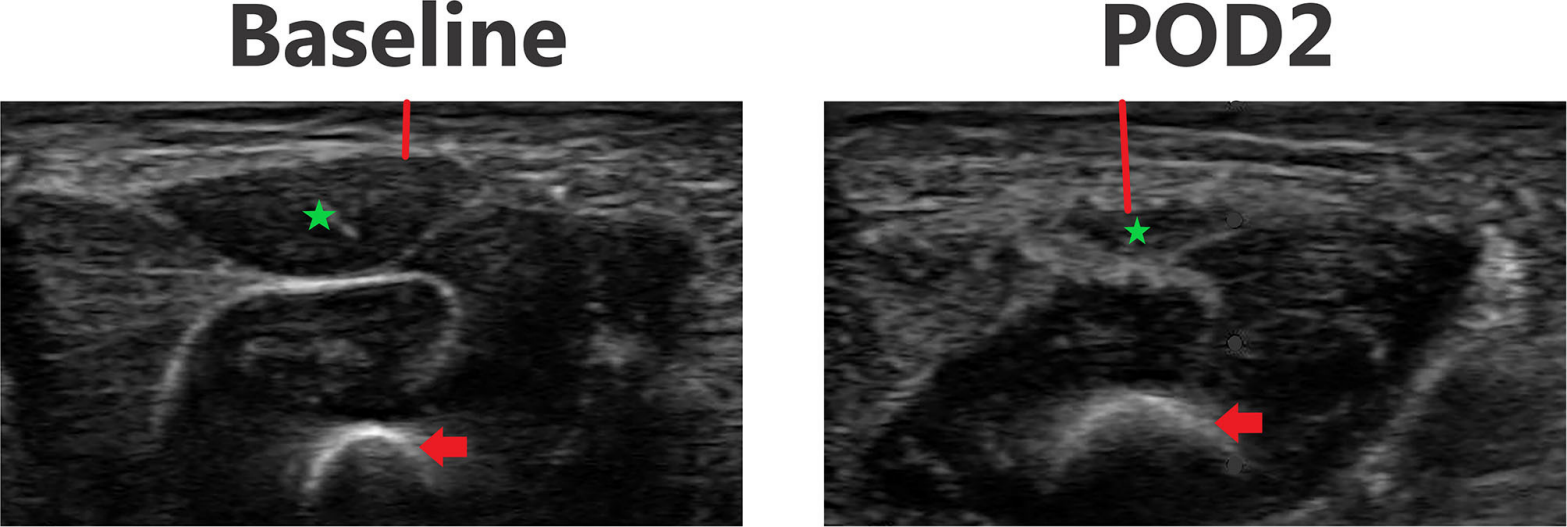
Point-of-care ultrasound images of anterior thigh at baseline and POD 2 of the same patient. Both images are presented on identical scales (total depth of 1.8 cm from the skin). Red vertical line demonstrates subcutaneous tissue thickness—there is a marked increase in subcutaneous tissue seen on POD 2. Red arrow—femur bone. Green star—echogenic muscle changes seen on POD 2.

Figures 3–6 provide examples of ultrasound images from each body site at baseline and POD 2 for the same patient and same lateral side. Each pair of images are presented on identical scales (depth noted in each figure caption). These are illustrative example of the changes seen post-operatively. There is a significant increase in subcutaneous tissue post-operatively, which is most pronounced in the lateral chest and lateral abdomen images. Qualitative changes in the muscle layers, specifically the muscle becoming more echogenic, is also noted and seen best in the lateral abdomen and anterior thigh images.

### Post-Operative Changes in Neonatal Surgical Subjects

**Figure 7:**
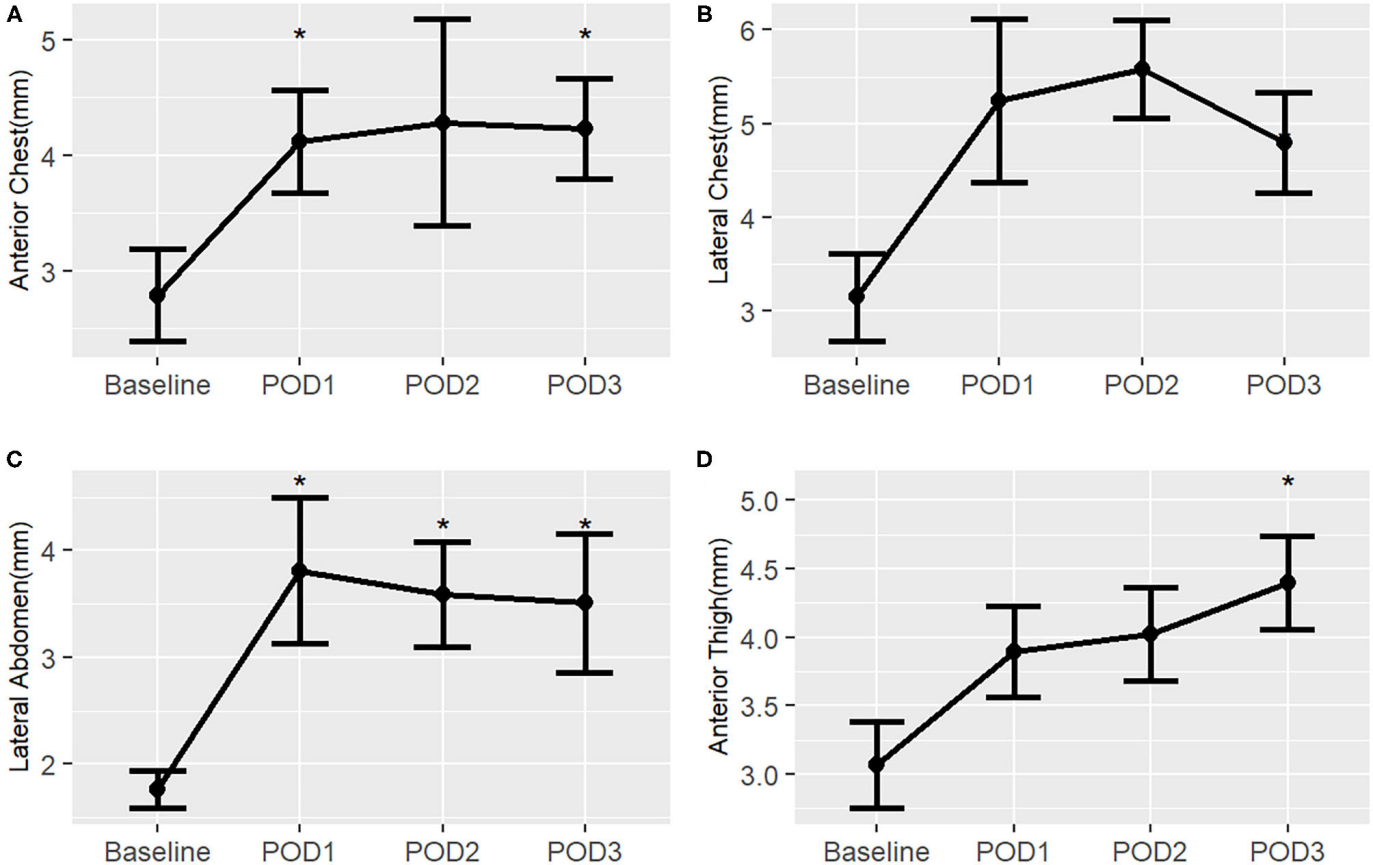
Post-operative changes in mean subcutaneous tissue thickness from baseline of surgical neonates, by right-sided body site. ^*^, *p* < 0.05. **(A)** Anterior chest; **(B)** Lateral chest; **(C)** Lateral abdomen; **(D)** Anterior thigh.

In the neonatal surgical group, there is a pattern of increased post-operative subcutaneous tissue thickness from baseline at all body sites suggesting extravascular fluid overload (Figure 7). Although the number of subjects was small in this validation study, the post-operative change in subcutaneous tissue thickness reached significance at multiple time points in several of the body sites. A difference from baseline is first noticeable by POD 1 and there is no evidence of substantial improvement by POD 3 for all body sites.

Surgical neonates with a post-operative open chest had a trend in the lateral abdomen for increased central skin edema compared to surgical neonates who did not have an open chest post-operatively (Supplementary Table 3). In those surgical neonates with a post-operative open chest, the increase in subcutaneous tissue thickness from baseline began to resolve by POD 5 but remained above baseline at all body sites.

### Post-Operative Changes in Infant Surgical Subjects

**Figure 8:**
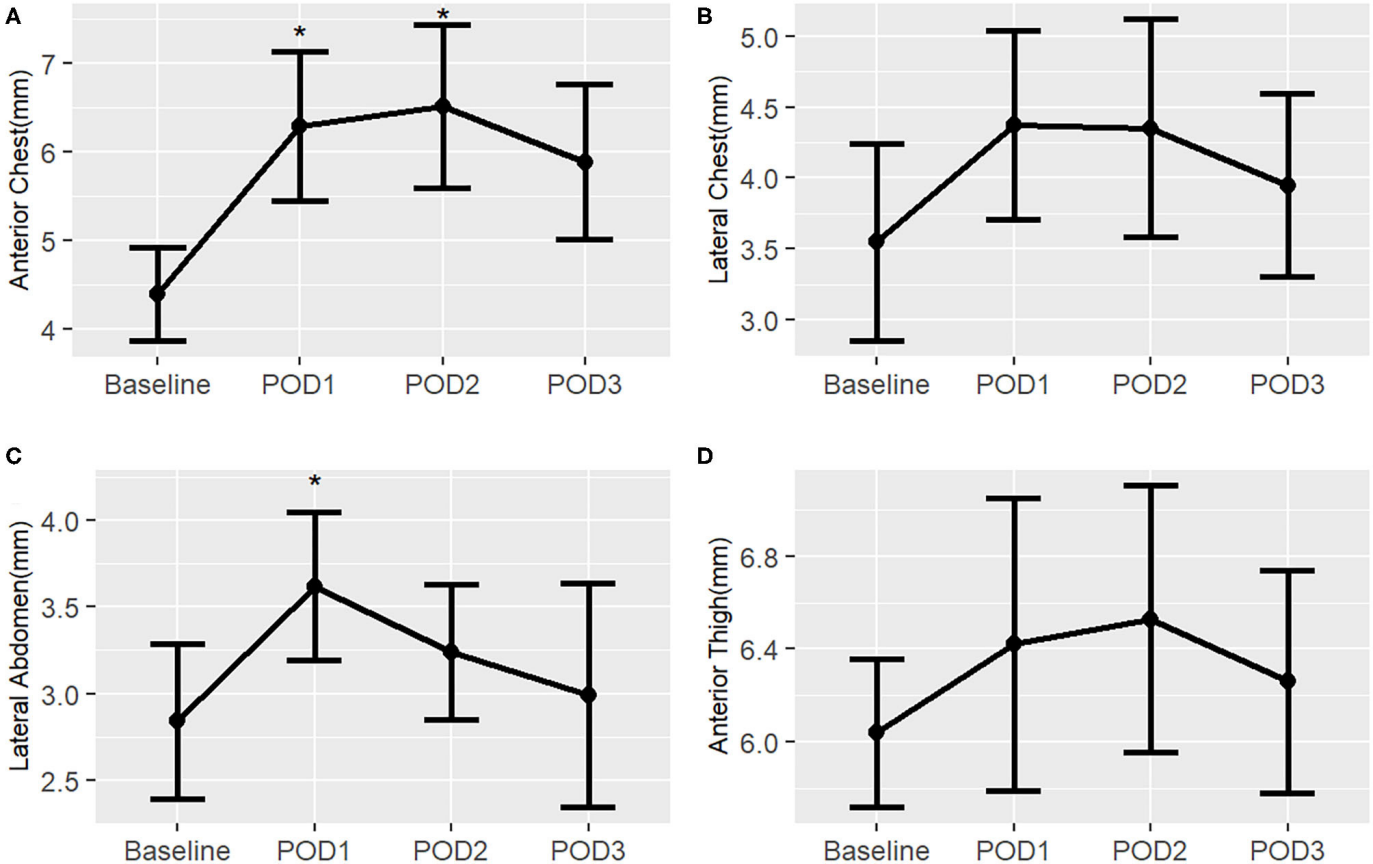
Post-operative changes in mean subcutaneous tissue thickness from baseline of surgical infants, by right-sided body site. ^*^, *p* < 0.05. **(A)** Anterior chest; **(B)** Lateral chest; **(C)** Lateral abdomen; **(D)** Anterior thigh.

In the surgical infant group, there is a pattern of increased subcutaneous tissue thickness post-operatively, but this trend is not as profound as in the neonatal surgical group (Figure 8). There was a statistically significant increase from baseline in the anterior chest on POD 1 and 2 as well as in the lateral abdomen on POD 1. There was a notable improvement to near-baseline by POD 3 in all body sites. Median measurement data is available in Supplementary Table 4.

### Inter- and Intra-User Variability

The overall inter-user variability had an ICC of 0.82 signifying that there was good correlation between the measurements obtained from the two ultrasound scanners at each body site. The overall intra-user variability had an ICC of 0.95, which demonstrates there was excellent correlation between the measurements obtained from the three images taken at each body site by a single operator. There was no notable difference in intra-user variability between ultrasound scans obtained at baseline or post-operatively. There was a better correlation between measurements from two ultrasound scanners on post-operative images than baseline images. Prior to the interim ultrasound training, the inter-user correlation was good (ICC 0.70). This inter-user correlation improved (ICC 0.87) following interim ultrasound training. All ICC results are presented in Table 3.

**Table 3:** Inter and intra-user variability.

	**Intra-user ICC** ^**a**^	**Inter-user ICC** ^**a**^
All	0.95 (0.946–0.954)	0.82 (0.83–0.87)
Baseline	0.94 (0.93–0.95)	0.75 (0.71–0.78)
Post-operative	0.93 (0.92–0.94)	0.82 (0.78–0.85)
Pre- and post-interim ultrasound training	n/a	Pre: 0.70 (0.65–0.74)
		Post: 0.87 (0.84–0.90)

a *ICC (95% confidence interval)*.

### Automated Measurement Correlation

**Figure 9:**
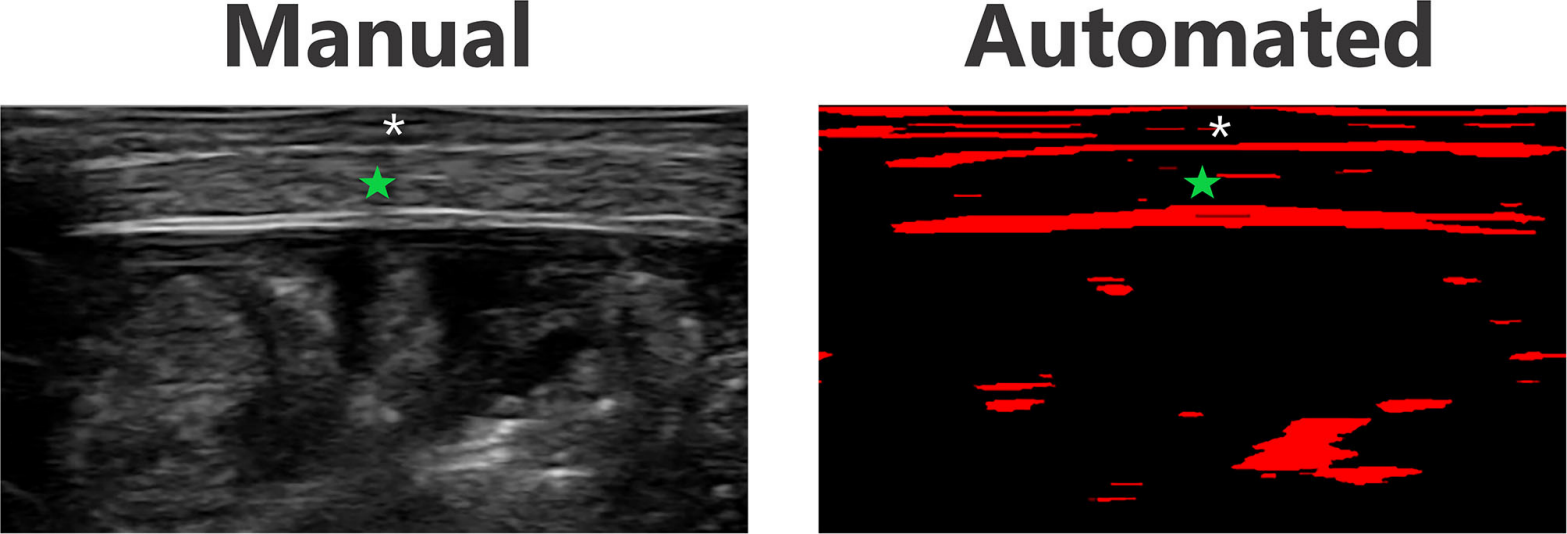
Ultrasound and automated MuscleSound, Inc. software image example of the lateral abdomen in the same subject. Green star—muscle layer. Asterisk—subcutaneous tissue layer.

An example of the MuscleSound, Inc. software image used as part of the automated measurement process is shown in Figure 9. Correlation between manual and automated baseline, measurements for right sided lateral abdomen and left sided anterior thigh of all subjects was strong (Table 4).

**Table 4:** Correlation between manual and automated baseline measurements.

	**Rho correlation (95% CI^**a**^)**	***P*** **-value**
Lateral abdomen (right side)	0.68 (0.52–0.79)	<0.0001
Anterior thigh (left side)	0.58 (0.38–0.72)	<0.0001

a *CI, confidence interval*.

## Discussion

Our study is the first to validate the use of point-of-care ultrasound to measure subcutaneous tissue thickness and postoperative edema in neonates and infants with CHD. At baseline, we found no significant differences between right and left body sides nor were there any significant differences between neonatal controls and surgical subjects. Post-operatively, acute increases in subcutaneous tissue thickness suggested the development of skin edema, consistent with extravascular volume overload. We found a pattern of increased skin edema in neonates post-operatively with a similar, but not as pronounced, trend in post-operative infants. All surgical subjects developed edema by POD 1. This extravascular fluid overload began to improve in infants after POD 1 but persisted in neonates through POD 3. Intra- and inter-user variability demonstrated good reproducibility of ultrasound measurements. Pilot automated baseline measurements of the lateral abdomen and anterior thigh had a good correlation to manual measurements. This study verified that point-of-care ultrasound is a novel, reproducible tool to quantify extravascular volume overload in neonates and infants with CHD.

The use of ultrasound to evaluate skin edema has not been studied in neonates or infants with CHD. Skin ultrasound is performed in both adult and pediatric emergency departments to diagnose skin and soft tissue infections, which can include qualitatively recognizing edema (32–34). However, the quantification of extravascular fluid overload via ultrasound has rarely been studied in adults and never previously attempted in infants with CHD (26, 35). Yanagisawa et al. used ultrasound to evaluate for skin edema in pregnant women and was able to manually measure the depth of subcutaneous edema (26). A study by Eisenbeiss et al. demonstrated that subcutaneous tissue thickness increased following fluid infusion and can be measured by ultrasound in healthy adult volunteers (35). Our study is the first to apply this ultrasound tool of measuring fluid overload to neonates and infants with CHD.

As expected, we found no major differences between left and right-sided body measurements demonstrating the consistency of skin ultrasound to measure subcutaneous tissue thickness. While there were two statistically significant differences, we suspect these are of minimal clinical significance given the small absolute degree of difference. More surprisingly, we did not identify consistent differences between neonates with CHD at baseline and healthy neonatal controls. We expected to find increased subcutaneous tissue in the healthy neonates as it is well-established that infants with CHD frequently struggle to achieve adequate somatic growth (36, 37). In fact, the only statistically significant difference that we did find was that neonates with CHD had a minimally increased subcutaneous tissue thickness, on the degree of 1 mm, in the lateral chest compared to controls, and this difference was not reproduced in any other body site. Therefore, we postulate that neonates with CHD may have some degree of baseline extravascular fluid overload and, as a result, appear to have comparable, to potentially increased, subcutaneous tissue thickness when compared to healthy neonates (38). A larger study is needed to better define the quality and quantity of subcutaneous tissue in neonates with CHD compared to healthy neonates.

While our study is the first to quantify post-operative extravascular fluid overload, the timing of extravascular volume overload found in our subjects is consistent with other markers of fluid overload in this population. Hassinger et al. showed that fluid overload occurred by POD 1 in 30% of the infants and older children who underwent cardiac surgery in their study (8). Interestingly, Hassinger et al. also noted that early fluid overload (development by POD 1) was more likely to occur in the younger patients, however it is important to point out that neonates were not included in this study so the incidence of early post-operative fluid overload may be even higher in the neonatal population (8). Our results support these data. We described the acute development of extravascular fluid overload post-operatively in both surgical neonates and infants, with a less pronounced trend in the infant group. By POD 3, infants had near-resolution of their extravascular fluid overload while surgical neonates had minimal improvement. Lex et al. demonstrated that fluid overload improved by the end of POD 2 in infants following cardiac surgery and younger patients, including newborns, remained fluid overloaded by the end of POD 2, which aligns with our findings (7). We hypothesize that this difference between infant and neonate fluid status may be related to several potential mechanisms including: (i) biologic differences as neonates have immature renal function that can limit fluid and sodium regulation; (ii) immunologic differences as the risk of capillary leak after cardiopulmonary bypass is higher in younger patients; and (iii) differences in surgical complexity as our neonates had higher STAT scores and more cases of delayed sternal closure than the infant group despite similar operative times (12, 39, 40).

There was minimal intra-user variability of the skin ultrasound in our study, confirming that there was high reliability between multiple ultrasound images taken by a single research team member. Inter-user reliability of the skin ultrasound, specifically variability between ultrasound images taken by different research team members, was good signifying high reproducibility, although slightly lower than the intra-user variability. Interestingly, we initially hypothesized that inter-user variability of baseline ultrasound images would be better than post-operative ultrasound images (due to expected distortion of landmarks by postoperative edema), yet we found the opposite. We offer two potential explanations for this unexpected finding: (i) the post-operative ultrasound may have been easier to perform with less subject movement as many subjects were intubated and/or sedated in the early post-operative period while baseline ultrasound scans were frequently performed on the awake, unsedated subject, (ii) the absolute difference in variability may have been the same between the baseline and post-operative ultrasound images, but the relative difference in variability could be made trivial in the post-operative ultrasound images due to the presence of significant skin edema.

Importantly, we demonstrated that overall inter-user variability improved following a second short ultrasound training and implementation of a visual hand-out. The ability to achieve high reproducibility of ultrasound images by minimally trained ultrasound operators who came from a variety of professional backgrounds exemplifies the potential of this tool to be used as bedside, point-of-care testing in the future. Furthermore, automated measurements of the ultrasound images would make this tool even more accessible for point-of-care use as manual measurements by a trained professional would not be required. Pilot testing of MuscleSound, Inc.'s automated measurement software was promising for this purpose as automated measurements correlated well with manual measurements of the baseline lateral abdomen and anterior thigh.

### Study limitations

Several limitations existed in our study. Most notably, our small sample size, especially in our surgical cohorts, impacted our ability to assess for small differences between groups and generalize this data to all neonates and infants with CHD. Similarly, the single center nature of the study limited the overall generalizability of the findings, especially as institutional differences in surgical practice, cardiopulmonary bypass, and post-operative management may impact the subject's fluid status. This study was powered to establish the reproducibility of skin ultrasound and demonstrate measurable change in subcutaneous tissue thickness post-operatively. Therefore, future research is needed to correlate these findings to clinical outcomes and generalize the results to the broader population. Technical challenges did exist, such as the use of a large ultrasound probe relative to the small size of the subject, unstable or unsedated subjects, and interfering lines/tubes. However, despite these issues, our intra- and inter-user variability was low, demonstrating the robust nature of this novel platform.

### Future directions

Important future steps in this line of research will be to further develop automated measurements at all body sites, including post-operative ultrasound images. We are hopeful that this point-of-care skin ultrasound paired with MuscleSound, Inc.'s automated measurement software can become a non-invasive, bedside tool to quantify a patient's extravascular fluid status. Additional study is needed to correlate our findings of extravascular fluid overload with standard markers of fluid overload, such as daily weights, fluid balance, and clinical assessment. This objective, novel method could be used to guide targeted post-operative therapies, including fluid administration and diuretic use, as well as aid in the timing of post-operative procedures such as delayed sternal closure and weaning from mechanical ventilation. As the long-term goal is to ultimately improve post-operative outcomes in infants and neonates with CHD, association of extravascular fluid overload measured by point-of-care ultrasound with clinical outcomes will be a critical future point of inquiry.

## Conclusions

Point-of-care skin ultrasound is a reproducible and reliable method to measure subcutaneous tissue in neonates and infants with and without CHD. Acutely increased subcutaneous tissue thickness is consistent with development of postoperative edema. All surgical subjects developed extravascular fluid overload by POD 1 and this began to improve in infants after POD 1 but persisted in neonates through POD 3. Point-of-care skin ultrasound is a non-invasive, bedside tool that could objectively measure extravascular fluid overload in neonates and infants with CHD. This represents a novel way to quantify extravascular volume overload with the potential to be translated to the bedside as point-of-care testing. Further research is needed to validate the use of automated measurement software and determine how skin edema measurements correlate to standard markers of fluid overload and clinical outcomes.

## Data Availability Statement

The raw data supporting the conclusions of this article will be made available by the authors, without undue reservation.

## Ethics Statement

The studies involving human participants were reviewed and approved by Colorado Multiple Institutional Review Board. Written informed consent to participate in this study was provided by the participants' legal guardian/next of kin.

## Author Contributions

JP and JD provided substantial contribution to conception and design of the research project and manuscript and drafted the manuscript and revised it critically for important intellectual content. JH provided substantial contribution to research implementation and data collection, including performing manual ultrasound measurements. LS performed the statistical analysis. All authors revised the article critically for important intellectual content and approved the final article as submitted and agree to be accountable for all aspects of the work.

## Conflict of Interest

The authors declare that the research was conducted in the absence of any commercial or financial relationships that could be construed as a potential conflict of interest.

## Publisher's Note

All claims expressed in this article are solely those of the authors and do not necessarily represent those of their affiliated organizations, or those of the publisher, the editors and the reviewers. Any product that may be evaluated in this article, or claim that may be made by its manufacturer, is not guaranteed or endorsed by the publisher.
