# Supplementary material for: Validation of Point-of-Care Ultrasound to Measure Perioperative Edema in Infants With Congenital Heart Disease
Source: Front Pediatr. 2021 Aug 23;9:727571. doi: 10.3389/fped.2021.727571 (PMC8419458; doi:10.3389/fped.2021.727571)
Supplement: Supplementary file 1 [file Table_1.docx]

**Supplementary Table 1: List of cardiac diagnoses of surgical subjects**

| **Neonatal surgical subjects** | |
| --- | --- |
| **Primary cardiac diagnosis** | **Number of subjects**  **(N = 12)** |
| Aortopulmonary window with interrupted aortic arch | 1 (8%)^a^ |
| Coarctation of the aorta | 1 (8%) |
| Complex single ventricle (example: heterotaxy) | 1 (8%) |
| Double outlet right ventricle with malposed great arteries and subpulmonic ventricular septal defect | 1 (8%) |
| D-transposition of the great arteries | 1 (8%) |
| Hypoplastic left heart syndrome | 3 (25%) |
| Interrupted aortic arch | 2 (17%) |
| Truncus arteriosus with coarctation of the aorta | 1 (8%) |
| Ventricular septal defect with coarctation of the aorta | 1 (8%) |
| **Infant surgical subjects** | |
| **Primary cardiac diagnosis** | **Number of subjects**  **(N = 10)** |
| Aortopulmonary window with interrupted aortic arch | 1 (10%)^a^ |
| Atrioventricular septal defect, complete | 2 (20%) |
| Hypoplastic left heart syndrome | 3 (30%) |
| Tetralogy of Fallot | 3 (30%) |
| Ventricular septal defect | 1 (10%) |

^a^ Categorical data are expressed as n (%).
