# Supplementary material for: Validation of Point-of-Care Ultrasound to Measure Perioperative Edema in Infants With Congenital Heart Disease
Source: Front Pediatr. 2021 Aug 23;9:727571. doi: 10.3389/fped.2021.727571 (PMC8419458; doi:10.3389/fped.2021.727571)
Supplement: Supplementary file 2 [file Table_2.docx]

**Supplementary Table 2: Mean baseline measurements of controls and surgical neonates, by body site.**

|  | **Control subjects** | **Neonatal surgical subjects** | **P-value** |
| --- | --- | --- | --- |
| **Anterior Chest^a^** (mm) | 2.5 (0.8) ^b^ | 2.9 (1.3) | 0.30 |
| **Lateral Chest^a^** (mm) | 2.0 (0.7) | 3.1 (1.4) | 0.02 |
| **Lateral Abdomen^a^** (mm) | 1.8 (0.7) | 1.8 (0.5) | 0.97 |
| **Anterior Thigh^a^** (mm) | 2.9 (0.9) | 3.2 (0.9) | 0.31 |

*mm = millimeters.* ^a^ Right-sided body site. ^b^ Continuous data are expressed as the mean (standard deviation).
