# Supplementary material for: Validation of Point-of-Care Ultrasound to Measure Perioperative Edema in Infants With Congenital Heart Disease
Source: Front Pediatr. 2021 Aug 23;9:727571. doi: 10.3389/fped.2021.727571 (PMC8419458; doi:10.3389/fped.2021.727571)
Supplement: Supplementary file 3 [file Table_3.docx]

**Supplementary Table 3: Comparison of post-operative median subcutaneous tissue thickness in surgical neonates with and without a post-operative open chest, by site.**

|  | **Anterior chest** | | | **Lateral chest** | | | **Lateral abdomen** | | | **Anterior thigh** | | |
| --- | --- | --- | --- | --- | --- | --- | --- | --- | --- | --- | --- | --- |
|  | **No open chest** | **Open chest** | **p-value** | **No open chest** | **Open chest** | **p-value** | **No open chest** | **Open chest** | **p-value** | **No open chest** | **Open chest** | **p-value** |
| **Baseline** (mm) | 1.7  (1.7-2.1)^a^ | 3.0  (1.8-4.4) | 0.09 | 2.7  (1.9-5.0) | 3.2  (1.6-3.7) | 1.0 | 1.4  (1.2-1.7) | 1.8  (1.5-2.0) | 0.11 | 3.0  (2.1-3.1) | 2.9  (2.0-2.7) | 1.0 |
| **POD1** (mm) | 3.5  (3.2-5.1) | 3.6  (2.5-5.9) | 0.82 | 4.9  (1.7-6.5) | 4.2  (3.7-9.0) | 0.90 | 1.8  (1.7-6.0) | 4.6  (2.2-5.2) | 0.65 | 3.9  (2.2-4.2) | 4.0  (3.2-5.0) | 0.55 |
| **POD2** (mm) | 2.9  (2.7-2.9) | 4.4  (2.0-5.8) | 0.36 | 3.0  (2.2-4.7) | 7.0  (4.7-7.0) | 0.10 | 1.8  (1.5-4.9) | 4.2  (3.0-5.2) | 0.25 | 4.2  (2.5-5.2) | 4.0  (3.1-5.1) | 0.86 |
| **POD3** (mm) | 3.2  (3.0-4.2) | 4.7  (3.0-5.7) | 0.42 | 4.3  (3.9-4.7) | 4.2  (3.5-7.2) | 1.0 | 1.8  (1.2-2.8) | 4.1  (2.5-4.8) | 0.11 | 4.2  (3.0-5.7) | 4.8  (4-5.1) | 1.0 |
| **POD4** (mm) | n/a | 5.0 (4.0-5.5) |  | n/a | 4.0 (4.0-6.9) |  | n/a | 2.62 (1.7-4.2) |  | n/a | 5.25 (4.5-5.83) |  |
| **POD5** (mm) | n/a | 3.21 (3.0-5.0) |  | n/a | 4.25 (2.6-5.6) |  | n/a | 2.0 (1.7-3.8) |  | n/a | 3.92 (3.0-5.4) |  |

*mm = millimeters.* ^a^ Continuous data are expressed as the median (interquartile range).
