# Supplementary material for: Validation of Point-of-Care Ultrasound to Measure Perioperative Edema in Infants With Congenital Heart Disease
Source: Front Pediatr. 2021 Aug 23;9:727571. doi: 10.3389/fped.2021.727571 (PMC8419458; doi:10.3389/fped.2021.727571)
Supplement: Supplementary file 4 [file Table_4.docx]

**Supplementary Table 4: Median post-operative subcutaneous tissue thickness in surgical infants, by site.**

|  | **Baseline** | **POD 1** | **POD 2** | **POD 3** |
| --- | --- | --- | --- | --- |
| **Anterior chest**^a^ (mm) | 4.3  (3.2-4.7)^b^ | 5.5  (4.7-8.0) | 5.7  (4.8-8.2) | 5.4  (4.1-6.3) |
| **Lateral chest**^a^ (mm) | 3.0  (1.8-3.9) | 3.7  (2.7-5.4) | 3.6  (2.3-5.6) | 3.0  (2.7-4.8) |
| **Lateral abdomen**^a^ (mm) | 3.1  (1.6-3.7) | 3.3  (3.0-3.9) | 2.8  (2.4-4.5) | 2.6  (2.3-4.2) |
| **Anterior thigh**^a^ (mm) | 6.0  (5.2-6.5) | 6.5  (5.6-7.9) | 6.5  (5.7-7.2) | 6.0  (5.2-7.6) |

*mm = millimeters.* ^a^ Right-sided body site. ^b^ Continuous data are expressed as the mean (standard deviation).
