# Supplementary material for: Validation of Point-of-Care Ultrasound to Measure Perioperative Edema in Infants With Congenital Heart Disease
Source: Front Pediatr. 2021 Aug 23;9:727571. doi: 10.3389/fped.2021.727571 (PMC8419458; doi:10.3389/fped.2021.727571)

<u>Ultrasound Instructions</u>

1. Positioning is key!
    a. Place the tablet and probe both near to the subject.
    b. Make sure you can comfortably hold the probe on the subject in one hand while using the tablet with your other hand.
        i. The key is to be able to use the probe and tablet at the same time without contorting your body position.
        ii. If you need to reach your body in order to press the tablet buttons, then you will likely move the probe by accident and distort the image.
2. Apply thin layer of gel onto probe
    a. Note that the thickness of the gel layer will affect the ultrasound measurements. The key is to be consistent in how much gel is used.
3. Where to place the probe
    a. Hold the probe like a pencil and place the probe gently on specified site.
        i. See below for specific site instructions
    b. Make sure the gray stripe on the probe is facing in the correct direction
        i. Either laterally or towards the feet, depending on the site
    c. Rest the heel of your hand on the subject.
        i. You do not need to apply pressure – in fact, pressing the probe into the skin can distort the skin layers we want to measure.
        ii. If the subject is moving, you may need someone to assist you in holding the subject still.
    d. Keep the probe perpendicular to the patient's body
4. Check your landmarks in the ultrasound image
    a. Site dependent – see below for specific site instructions
    b. General goals:
        i. Position the landmarks in the middle of the image
        ii. Keep skin layers in a straight horizontal line across the image.
    c. Try to make the ultrasound image appear similar to the gold standard image
5. If you do not see the landmarks:
    a. Confirm your probe positioning
    b. Try moving the probe left/right or up/down
    c. Try tilting the probe slightly up and down
6. Make sure you are still holding the probe gently on the skin
    a. Do NOT press the probe into the skin!
7. Without moving your hand holding the probe, freeze the image on the tablet by pressing the snowflake button with your other hand.
8. Take 3 images at this body site.

Specific Site Instructions

Anterior Chest

1) Positioning:
   a. Place the probe on the anterior upper chest, about 1cm above the nipple at the mid-clavicular line
   b. Make sure the gray stripe on the probe faces LATERAL
2) Landmarks:
   a. The pectoral muscle should appear as a black horizontal stripe across the image.
   b. There should be a bright line beneath the pectoral muscle that represents the boundary between the superficial tissue and the thoracic cavity.
   c. Position the muscle so it is as horizontal as possible; with the straightest portion in the middle of the image.
      i. This may be difficult due to the curvature of the chest wall
3) Gold standard images:

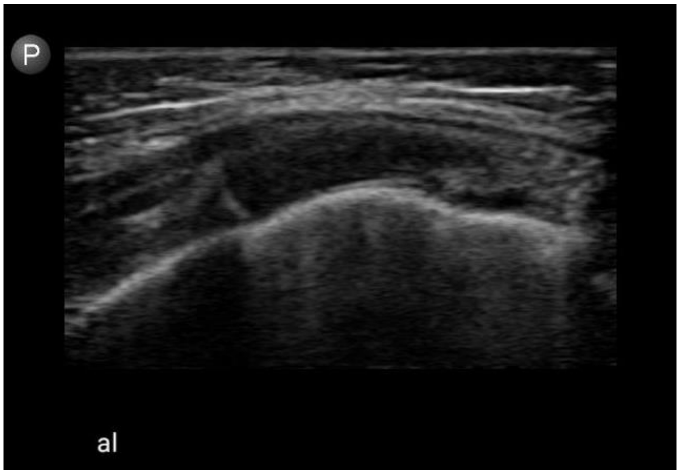

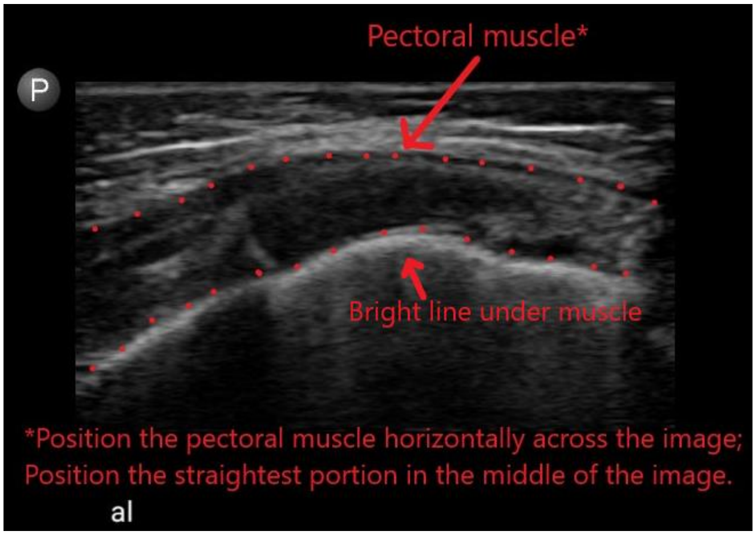

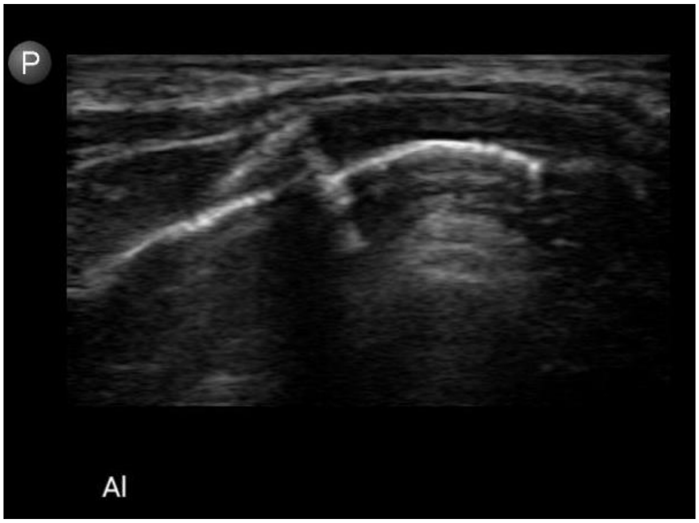

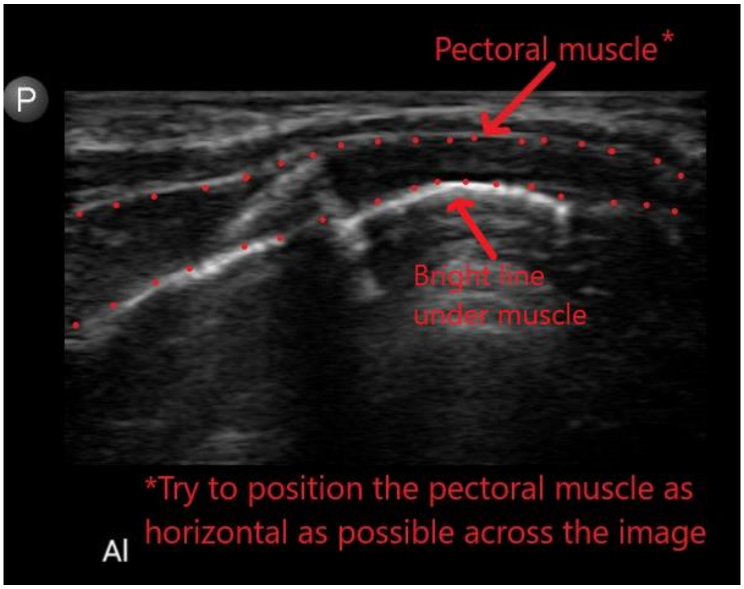

Lateral Chest

1) Positioning:
    a. Place the probe on the side of the chest, at the level of the nipple at the mid-axillary line
    b. Make sure the gray stripe on the probe faces the FEET
2) Landmarks:
    a. The ribs (bright white) should be seen in the middle of the image. Try to align a single rib so it is in the middle of the image and the top of the rib is horizontal across the image.
    b. There should be a dark shadow below each rib.
3) Gold standard images:

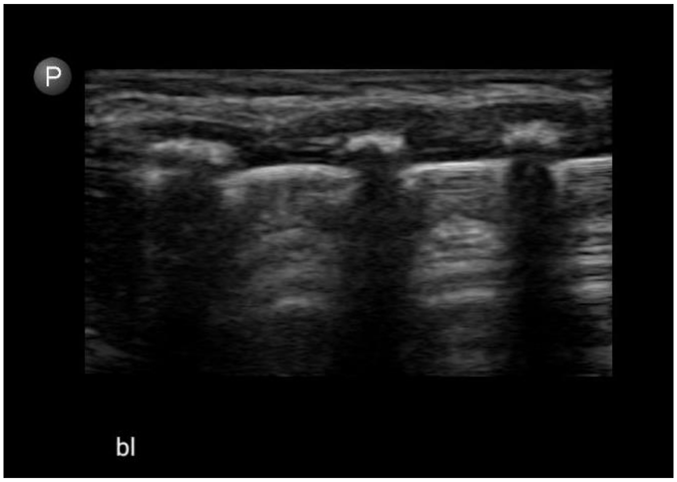

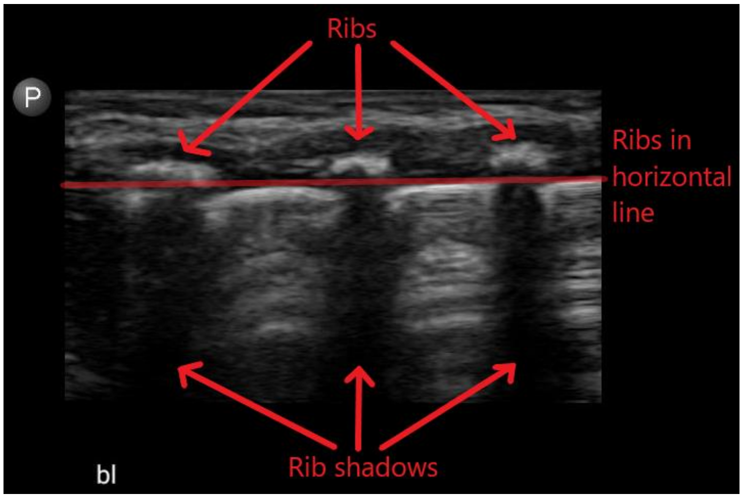

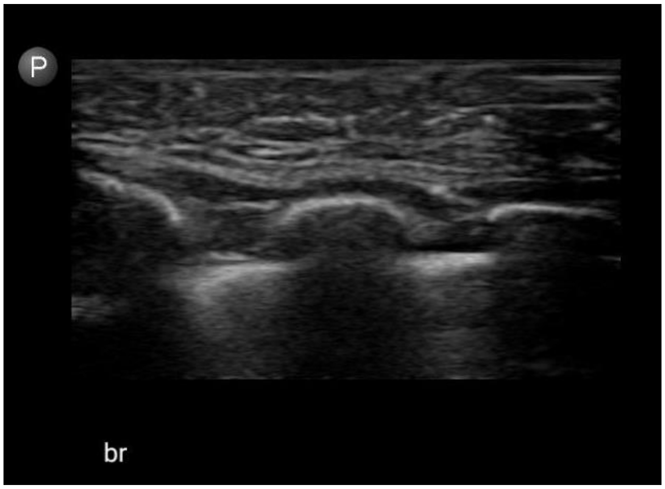

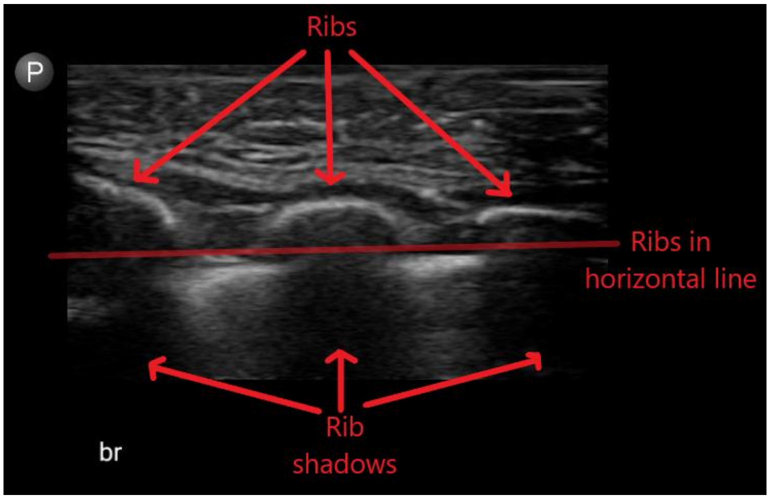

Lateral Abdomen

1) Positioning:
    a. Place the probe on the side of the abdomen, at the level of the umbilicus at the mid-axillary line
    b. Make sure the gray stripe on the probe faces the FEET
2) Landmarks:
    a. The liver (which will look dark grey and spongy) should be seen in the bottom half of the image.
    b. There should be skin layers above the liver; keep these skin layers in a straight horizontal line on the top of the image.
3) Gold standard images:

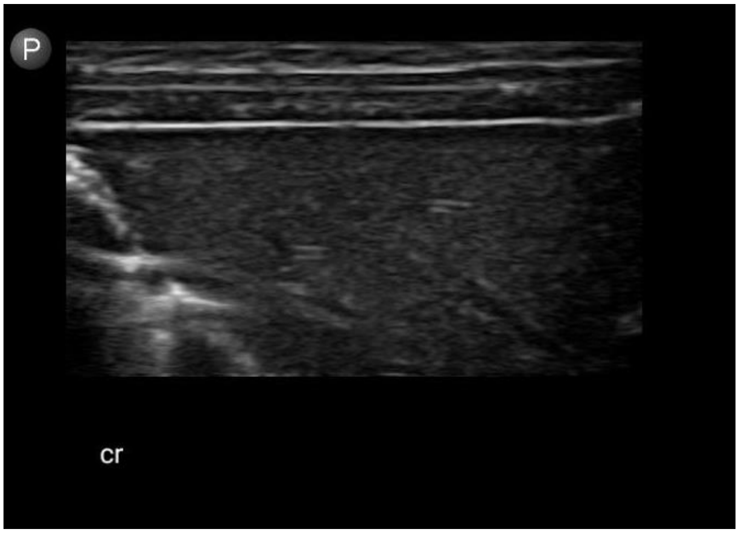

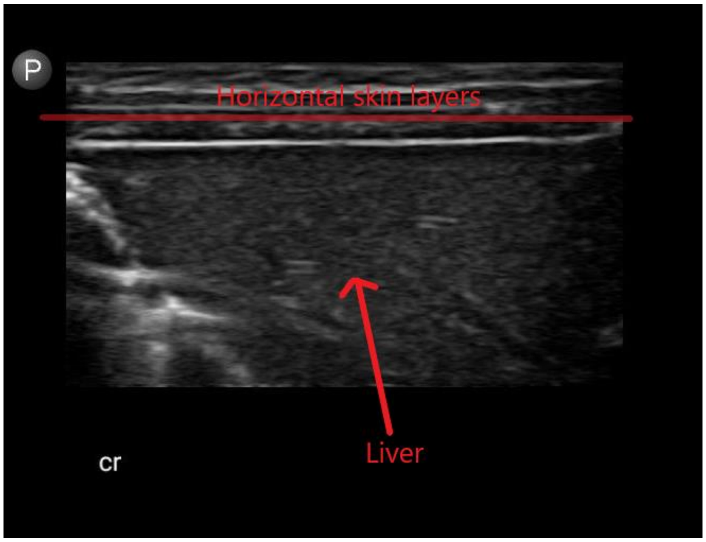

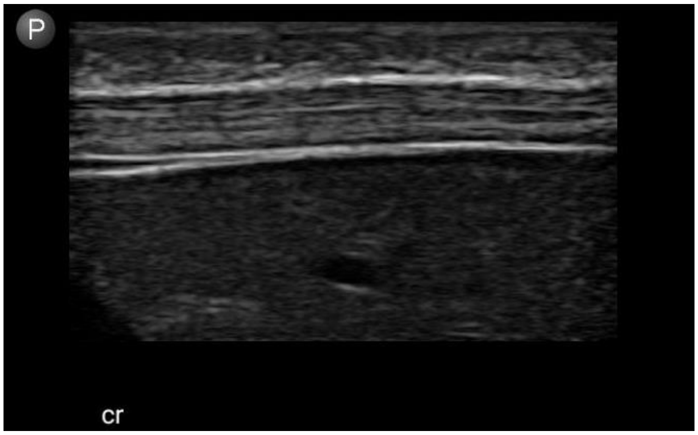

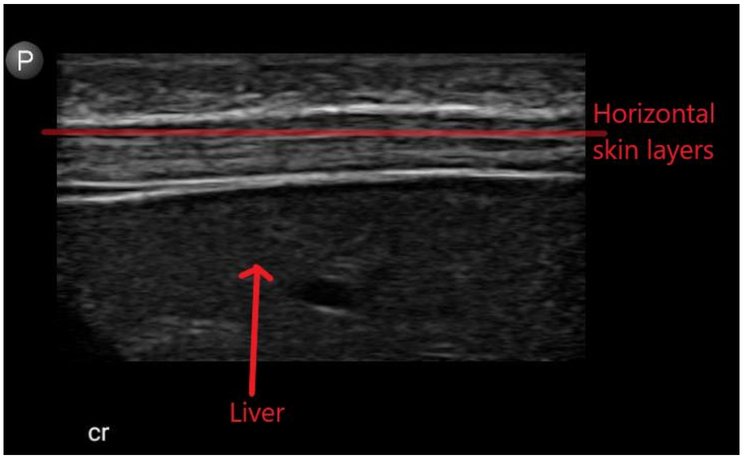

Anterior Thigh

1) Positioning:
    a. Place the probe in the middle of the anterior thigh, about 2cm above the patella (knee)
    b. Make sure the gray stripe on the probe faces LATERAL
2) Landmarks:
    a. The femur (bright white) should be seen in the bottom middle of the image
    b. The rectus femoris (RF) muscle (grey trapezoid) should be above the femur in the middle of the image. Ideally, there will be a white line all the way around the muscle.
    c. There should be three trapezoidal muscles surrounding the rectus femoris muscle and separating it from the femur. These muscles are called: vastus lateralis (VL), vastus medialis (VM), and vastus intermedius (VI).
    d. Keep the skin layers at the top of the images in a straight horizontal line
3) Gold standard images:

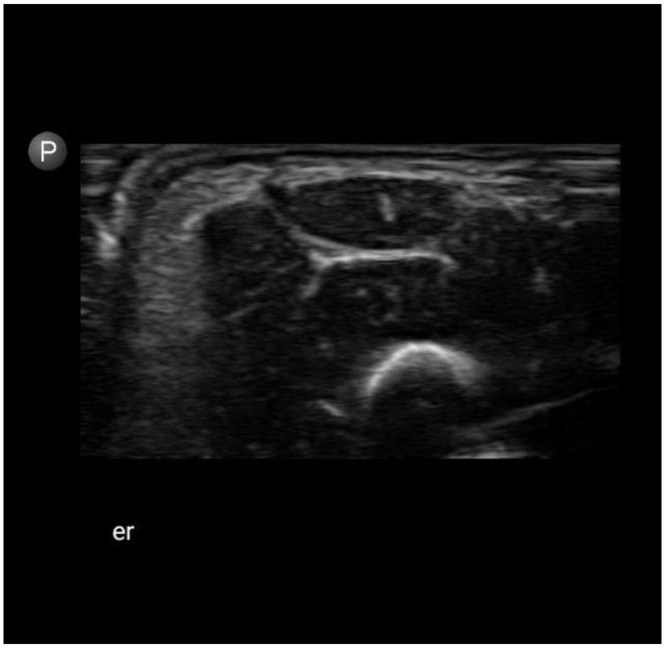

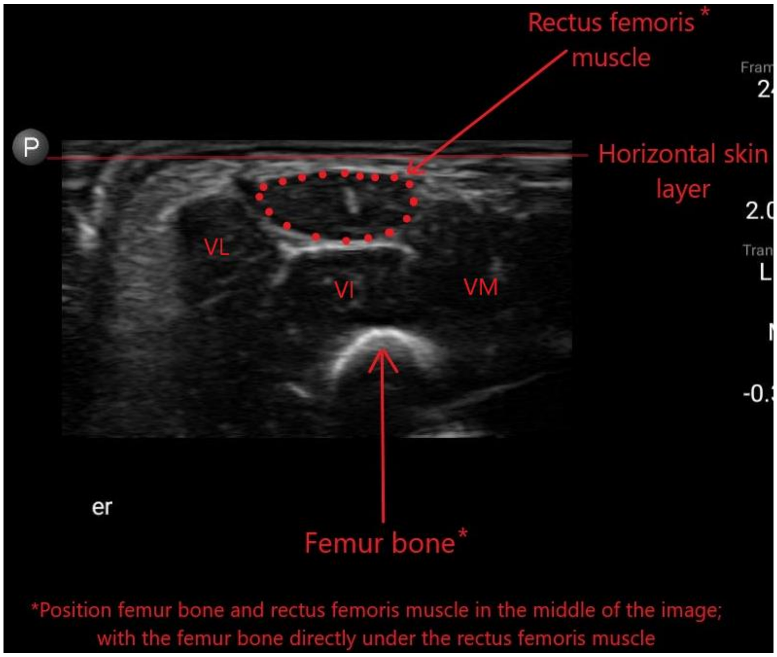

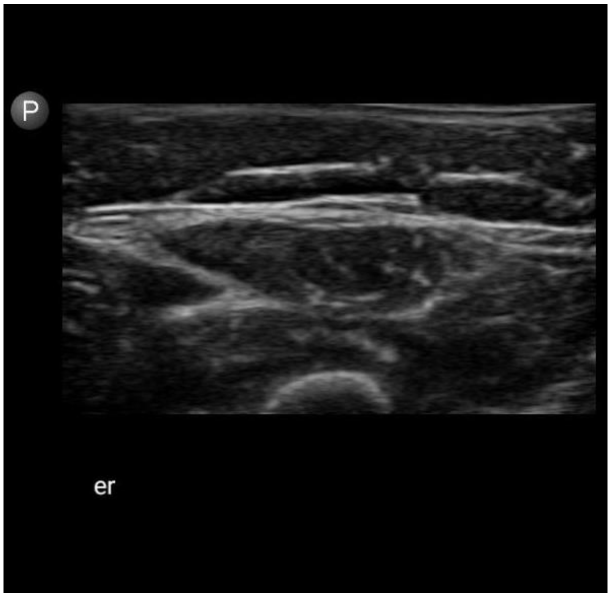

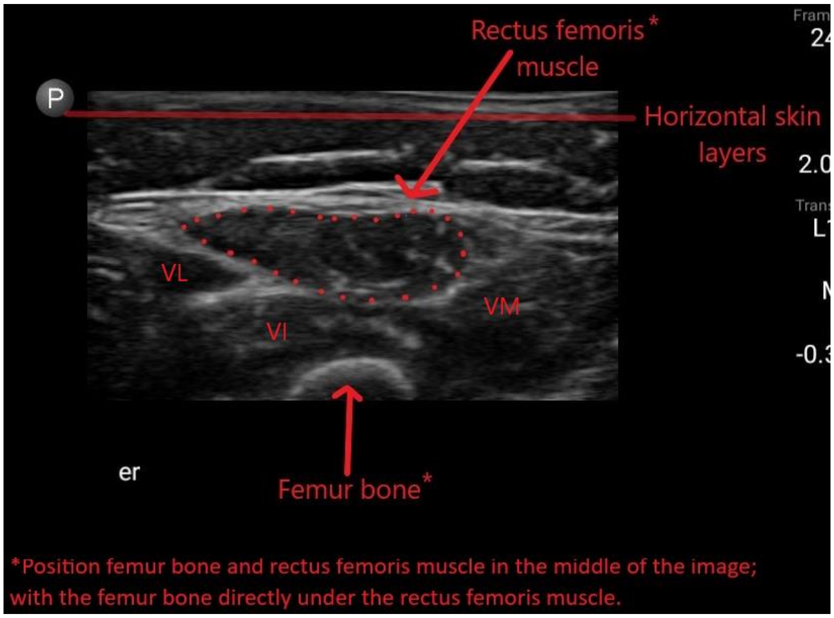

Supplement: Supplementary file 5 [file Data_Sheet_1.pdf]
